# Age-related changes in corneal parameters in the Turkish population: a Pentacam-based study

**DOI:** 10.1186/s12886-026-05365-9

**Published:** 2026-09-23

**Authors:** Denizcan Özizmirliler, İsmet Durak

**Affiliations:** 1 Department of Ophthalmology, Dr. Nevruz Erez State Hospital, Iğdır, Turkey; 2 Department of Ophthalmology, Dokuz Eylül University, Izmir, Turkey

**Keywords:** Cornea, Corneal topography, Pentacam

## Abstract

**Purpose:**

To evaluate anterior and posterior corneal surfaces and anterior segment parameters in the Turkish population using Pentacam, and to analyze age-related changes.

**Methods:**

This retrospective study included healthy participants aged 3–86 years who underwent Pentacam imaging at Dokuz Eylül University Faculty of Medicine. Participant-level descriptive summaries were based on right-eye measurements, whereas inferential analyses incorporated both eyes using linear mixed-effects models with a participant-level random intercept. Age was evaluated both categorically (3–19, 20–39, 40–59, and ≥ 60 years) and continuously using centered linear and quadratic age terms.

**Results:**

A total of 1202 eyes of 601 participants (49.1% male, 50.9% female; mean age 35.25 ± 23.02 years) were included. Age-group effects were supported for anterior K2, Kmean, Rflat, Rsteep, Rmean, anterior astigmatism magnitude and asphericity, posterior K1, posterior astigmatism magnitude and asphericity, thinnest corneal point, corneal volume, anterior chamber volume, and anterior chamber depth (nominal *p* < 0.05). Age effects for anterior K1, posterior K2, posterior Rsteep, and anterior and posterior steep astigmatism axes were laterality-dependent. Continuous-age models demonstrated quadratic trajectories for anterior K1, K2, Kmean, Rflat, Rsteep, Rmean, and anterior asphericity, with approximate turning points between 32 and 46 years. Thinnest corneal point, corneal volume, anterior chamber volume, and anterior chamber depth decreased with increasing age; the age slopes for anterior chamber volume and depth differed by sex.

**Conclusions:**

Pentacam-derived corneal and anterior segment parameters show non-uniform age-related variation. The most consistent changes involved anterior corneal curvature and shape, corneal and anterior chamber dimensions, whereas several posterior corneal and astigmatic-axis measures were laterality-dependent. These findings support age-aware interpretation of corneal tomography rather than a single uniform aging pattern.

**Supplementary Information:**

The online version contains supplementary material available at https://doi.org/10.1186/s12886-026-05365-9.

## Introduction

Corneal refractive power is determined by the parameters of the anterior and posterior corneal surfaces, along with corneal thickness. As with many other organs, aging leads to changes in corneal parameters. Understanding age-related changes in the cornea across different populations is essential for distinguishing between pathological and physiological alterations. Corneal parameters are crucial in evaluating ectatic corneal disorders, which are characterized by progressive structural deformation, and in determining the optimal treatment. Additionally, corneal thickness serve as an important parameter in the diagnosis of glaucoma [1]. A review of the literature reveals age-related changes in corneal thickness, corneal asphericity, as well as anterior and posterior astigmatism values and their axes across different populations [2–6]. Determining age-related changes in these parameters and understanding the overall shape of the cornea enhance the success of various ocular surgeries, particularly refractive surgery. Recognizing the influence of posterior corneal astigmatism on total corneal astigmatism has highlighted the importance of corneal parameters in calculating and implanting intraocular lens power during cataract surgery [7–11].

In cases where posterior corneal astigmatism cannot be directly measured, estimating PCA based on parameters such as age, sex, or other biometric factors may significantly reduce toric intraocular lens calculation errors. The aim of our study is to determine the anterior and posterior corneal surface parameters, as well as anterior segment parameters, in the Turkish population using the Pentacam (Oculus Optikgeräte GmbH, Wetzlar, Germany) topography device and to analyze the age-related variations in these parameters.

## Materials and methods

A detailed refractive examination and biomicroscopic evaluation were performed on all patients who presented to the Ophthalmology Clinic of Dokuz Eylül University Faculty of Medicine. Uncorrected visual acuity and best-corrected visual acuity (BCVA) values were recorded. A routine fundus examination was conducted. Data from individuals who underwent corneal topography measurements using the high-resolution Scheimpflug imaging system, the Pentacam corneal topography device, and who had no detected pathology were analyzed. This study was approved by the Ethical Committee of Dokuz Eylül University Faculty of Medicine (2021/36 − 11) and was conducted in accordance with the ethical principles stated in the Declaration of Helsinki and local regulations.

The inclusion criteria were defined as follows: completion of a comprehensive ophthalmologic examination, acquisition of corneal topography, being over the age of 3 years, and having no history of surgery or disease that could alter the anatomical structure of the cornea. The exclusion criteria included the presence of any ocular pathology other than refractive errors, a history of ocular disease, or a history of ocular surgery. Patients who met these criteria and presented to the clinic between October 1, 2021, and December 31, 2022, were included in the study.

Each morning, the topography device was calibrated. Corneal topography measurements were conducted between 08:30 AM and 5:00 PM by two different technicians. The same protocol was followed for all patients during the scanning process. Patients were positioned with their chins resting on the chin rest and their foreheads on the forehead strap, ensuring that their heads were completely horizontal. The room lights were turned off during image acquisition. Before the scan, patients were instructed to blink several times to prevent aberrations caused by tear film irregularities and to focus on the target at the center of the blue fixation light. Measurements were automatically performed using the Pentacam corneal topography device once the image was properly focused and the corneal apex was correctly aligned. After all measurements, error margins were checked, and only the highest-quality image data were included in the study.

Patients were categorized into four age groups: 3–19 years, 20–39 years, 40–59 years, and 60 years and above (60+). Corneal parameter values were analyzed according to these age groups, and age-related changes in these parameters were investigated. The keratometric values of the anterior and posterior corneal surfaces (K1, K2, and Kmean), astigmatism and its axes, the axes of the anterior and posterior corneal surfaces, radii of curvature (Rflat, Rsteep, Rmean), asphericity values (Q value), the thinnest value of the cornea, corneal volume, anterior chamber volume (ACV), anterior chamber depth (ACD), and ophthalmologic examination findings were retrospectively analyzed.

## Statistical analysis

**Fig. 1:**
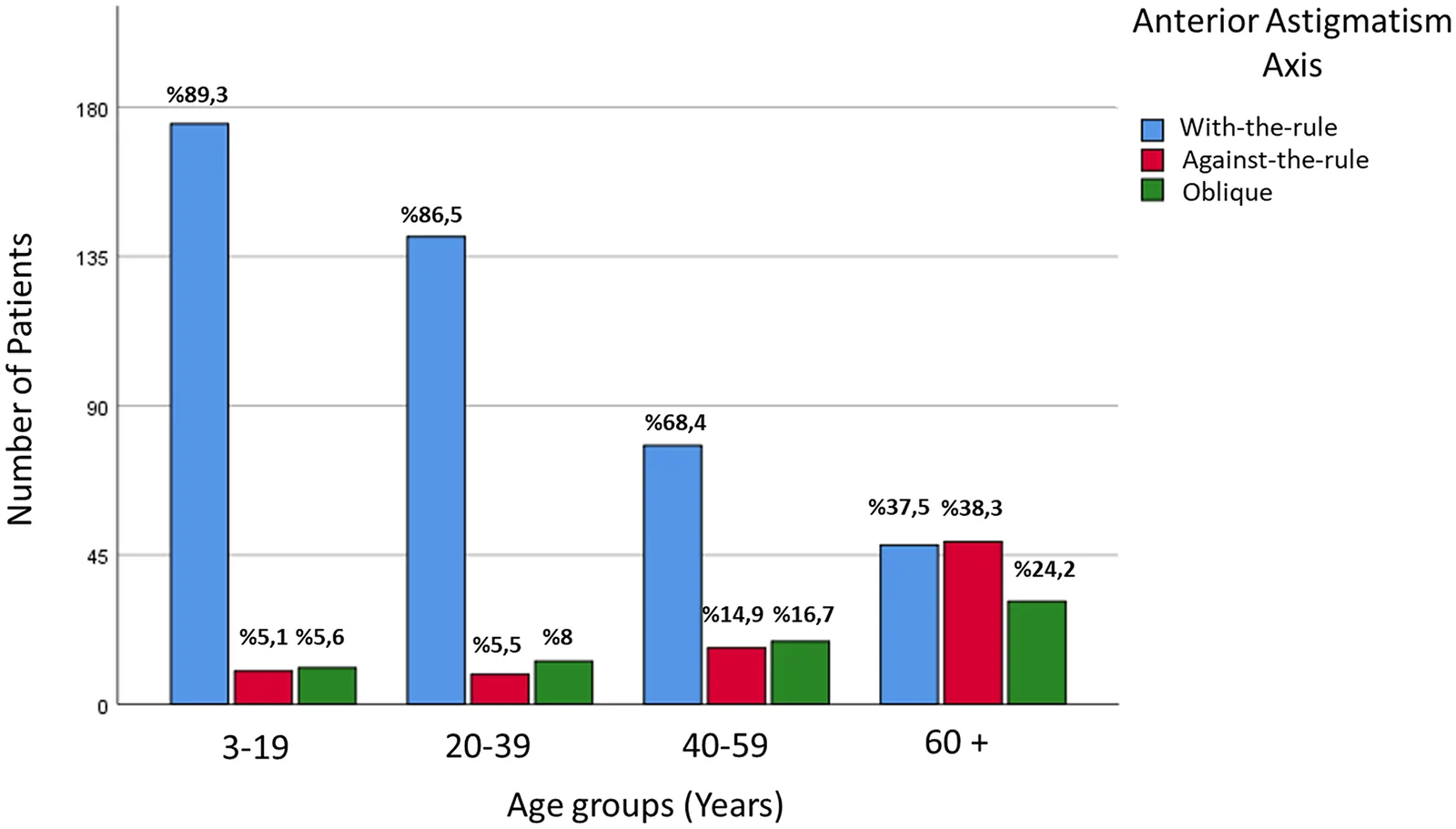
Raw participant-level right-eye distribution of anterior corneal astigmatism orientation across age groups. Bars show n (%). WTR: steep axis 60°–120°; ATR: 0°–30° or 150°–180°; oblique: 31°–59° or 121°–149°. Age-group denominators were 196, 163, 114, and 128 participants, respectively. The figure is descriptive and does not represent model-derived estimates

**Fig. 2:**
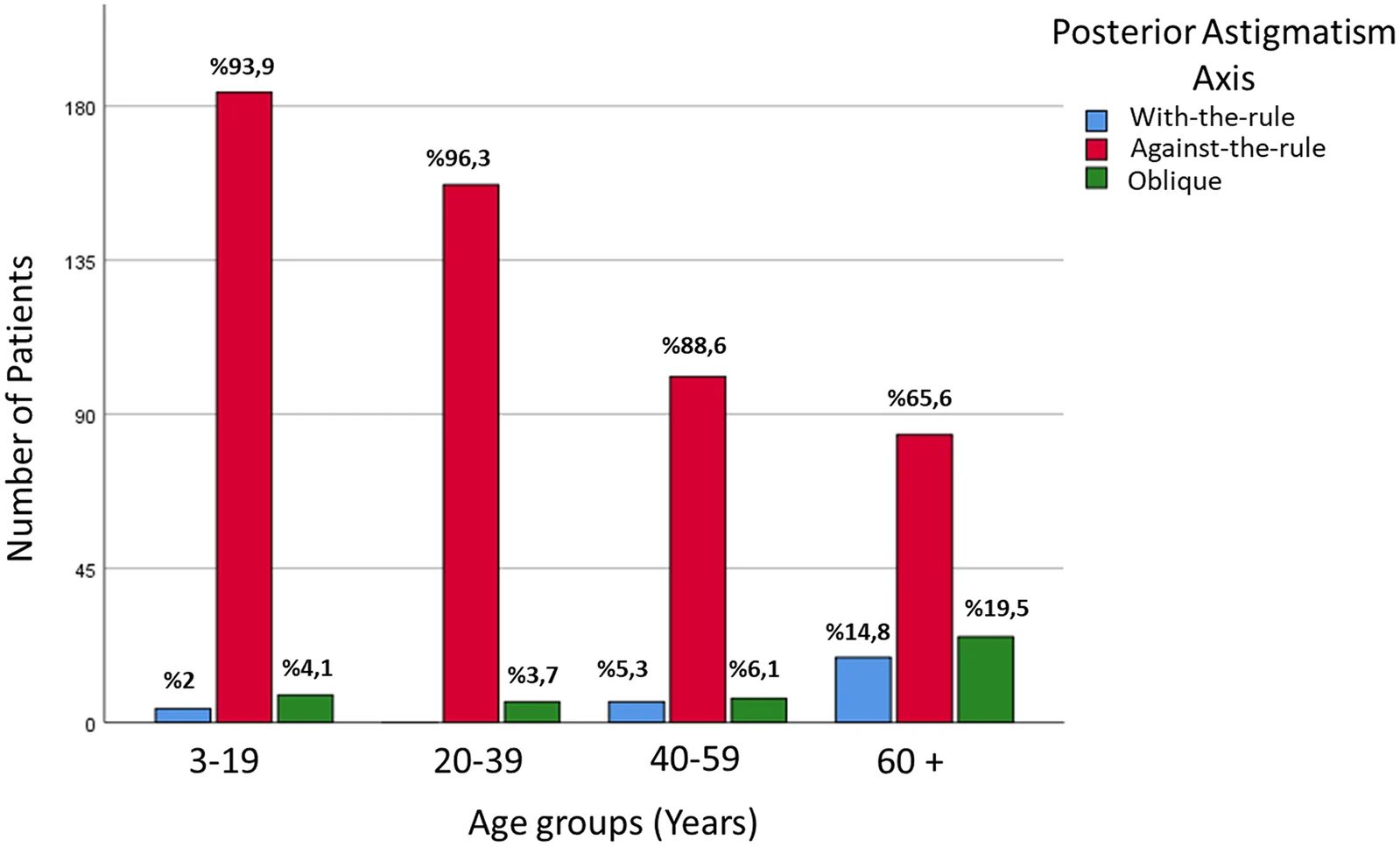
Raw participant-level right-eye distribution of posterior corneal astigmatism orientation across age groups. Bars show n (%). WTR: steep axis 60°–120°; ATR: 0°–30° or 150°–180°; oblique: 31°–59° or 121°–149°. Age-group denominators were 196, 163, 114, and 128 participants, respectively. The figure is descriptive and does not represent model-derived estimates

Descriptive statistics were presented as mean ± standard deviation (SD) for approximately normally distributed continuous variables and as median (minimum–maximum) for variables designated non-normal in the original analysis. Categorical variables were summarized as frequencies and percentages. Tables 1, 2 and 3; Figs. 1 and 2 used right-eye measurements only (one observation per participant). Inferential analyses were performed at the eye level and incorporated both eyes. For each outcome, a participant-level random-intercept linear mixed-effects model was fitted by restricted maximum likelihood (REML), implying compound symmetry for fellow-eye measurements; no additional residual covariance structure was specified. Fixed effects in the categorical-age models were age group, sex, laterality, age group × sex, age group × laterality, and sex × laterality. All prespecified interactions were retained to preserve model hierarchy; no automated model-selection procedure was used. Omnibus fixed effects were evaluated with Wald tests. When an interaction involving the factor of interest was significant, a single overall main-effect p value was not interpreted; instead, model-based simple effects and estimated marginal means with 95% confidence intervals were reported in Supplementary Table S2. No age-group pairwise comparisons were retained in the final analysis.

**Table 1:** Pentacam parameters by sex: participant-level right-eye summaries with mixed-model inference

Parameter	Male (*n* = 295)	Female (*n* = 306)	Overall sex effect *p*	Relevant significant interaction
Anterior K1-flat (D)	42.47 ± 1.50	43.05 ± 1.42	—	Sex×laterality *p* = 0.048
Anterior K2-steep (D)	43.64 ± 1.54	44.10 ± 1.53	< 0.001	None
Anterior Kmean (D)	43.05 ± 1.46	43.57 ± 1.43	< 0.001	None
Anterior Rflat (mm)	7.96 ± 0.29	7.85 ± 0.26	—	Sex×laterality *p* = 0.022
Anterior Rsteep (mm)	7.74 ± 0.27	7.66 ± 0.26	< 0.001	None
Anterior Rmean (mm)	7.84 ± 0.30	7.75 ± 0.25	< 0.001	None
Anterior astigmatism (D)	1.00 (0.00–5.30)	0.90 (0.00–4.00)	0.201	None
Anterior steep astigmatism axis (°)	94.50 (0.10–179.00)	88.55 (3.10–177.50)	—	Sex×laterality *p* = 0.008
Anterior asphericity (Q)	-0.32 (-0.74–1.14)	-0.35 (-0.99–0.27)	0.019	None
Posterior K1-flat (D)	-6.10 (-6.80–-4.70)	-6.20 (-6.90–-5.50)	< 0.001	None
Posterior K2-steep (D)	-6.40 (-7.30–-5.80)	-6.50 (-7.60–-5.80)	0.002	None
Posterior Kmean (D)	-6.30 (-6.90–-5.20)	-6.30 (-7.00–-5.60)	< 0.001	None
Posterior Rflat (mm)	6.56 (5.87–8.42)	6.45 (5.80–7.30)	< 0.001	None
Posterior Rsteep (mm)	6.21 ± 0.27	6.15 ± 0.25	< 0.001	None
Posterior Rmean (mm)	6.38 ± 0.26	6.31 ± 0.23	< 0.001	None
Posterior astigmatism (D)	0.30 (0.00–1.40)	0.30 (0.00–1.20)	—	Sex×laterality *p* = 0.033
Posterior steep astigmatism axis (°)	96.30 (3.20–169.60)	91.90 (7.90–173.60)	—	Sex×laterality *p* = 0.001
Posterior asphericity (Q)	-0.39 ± 0.18	-0.39 ± 0.17	0.406	None
Thinnest corneal point (µm)	549.94 ± 36.93	545.98 ± 33.26	0.154	None
Corneal volume (mm³)	61.23 ± 3.98	61.21 ± 3.76	—	Sex×laterality *p* = 0.050
Anterior chamber volume (mm³)	170.85 ± 44.42	163.13 ± 43.53	< 0.001	None
Anterior chamber depth (mm)	3.06 ± 0.46	3.04 ± 0.52	0.146	None

Note: Descriptive summaries use the right eye only (one observation per participant). Normally distributed variables are mean ± SD; variables designated non-normal in the original analysis are median (minimum–maximum). Inferential models include both eyes. When a significant interaction involving sex was present, no single overall sex p value was interpreted. All p values are nominal

**Table 2:** Pentacam parameters across age groups: participant-level right-eye summaries with mixed-model inference

Parameter	3–19 (*n* = 196)	20–39 (*n* = 163)	40–59 (*n* = 114)	≥ 60 (*n* = 128)	Overall age-group *p*	Relevant significant interaction
Anterior K1-flat (D)	42.83 ± 1.37	42.52 ± 1.46	42.59 ± 1.50	43.14 ± 1.62	—	Age group×laterality *p* = 0.045
Anterior K2-steep (D)	44.09 ± 1.45	43.59 ± 1.53	43.63 ± 1.60	44.12 ± 1.60	< 0.001	None
Anterior Kmean (D)	43.45 ± 1.36	43.06 ± 1.45	43.10 ± 1.47	43.63 ± 1.57	< 0.001	None
Anterior Rflat (mm)	7.89 ± 0.25	7.95 ± 0.28	7.93 ± 0.28	7.83 ± 0.30	0.001	None
Anterior Rsteep (mm)	7.66 ± 0.25	7.75 ± 0.27	7.75 ± 0.28	7.66 ± 0.28	< 0.001	None
Anterior Rmean (mm)	7.77 ± 0.29	7.85 ± 0.26	7.84 ± 0.27	7.75 ± 0.28	< 0.001	None
Anterior astigmatism (D)	1.10 (0.10–4.20)	0.90 (0.00–3.40)	0.70 (0.10–5.30)	0.80 (0.00–4.70)	< 0.001	None
Anterior steep astigmatism axis (°)	93.70 (11.80–179.00)	93.10 (6.70–176.80)	91.75 (2.80–177.50)	68.95 (0.10–177.90)	—	Age group×laterality p = < 0.001
Anterior asphericity (Q)	-0.39 (-0.78–-0.07)	-0.31 (-0.70–0.27)	-0.30 (-0.77–1.14)	-0.30 (-0.99–-0.03)	< 0.001	None
Posterior K1-flat (D)	-6.20 (-6.70–-4.70)	-6.10 (-6.80–-5.30)	-6.20 (-6.80–-5.50)	-6.20 (-6.90–-5.40)	0.029	None
Posterior K2-steep (D)	-6.50 (-7.30–-5.90)	-6.50 (-7.00–-5.90)	-6.40 (-7.60–-5.80)	-6.40 (-7.30–-5.80)	—	Age group×laterality *p* = 0.022
Posterior Kmean (D)	-6.30 (-6.90–-5.20)	-6.30 (-6.90–-5.60)	-6.30 (-6.90–-5.60)	-6.30 (-7.00–-5.60)	0.217	None
Posterior Rflat (mm)	6.46 (5.93–8.42)	6.52 (5.85–7.51)	6.49 (5.87–7.30)	6.47 (5.80–7.44)	0.084	None
Posterior Rsteep (mm)	6.13 ± 0.26	6.20 ± 0.23	6.21 ± 0.29	6.21 ± 0.27	—	Age group×laterality *p* = 0.038
Posterior Rmean (mm)	6.32 ± 0.24	6.36 ± 0.22	6.36 ± 0.27	6.34 ± 0.27	0.236	None
Posterior astigmatism (D)	0.30 (0.00–1.40)	0.30 (0.00–0.80)	0.30 (0.00–1.20)	0.20 (0.00–0.90)	< 0.001	None
Posterior steep astigmatism axis (°)	91.05 (11.40–163.40)	93.10 (55.10–139.20)	97.50 (24.90–173.60)	99.25 (3.20–167.80)	—	Age group×laterality p = < 0.001
Posterior asphericity (Q)	-0.34 ± 0.15	-0.35 ± 0.15	-0.45 ± 0.18	-0.48 ± 0.18	< 0.001	None
Thinnest corneal point (µm)	558.52 ± 32.91	543.35 ± 33.06	545.64 ± 31.86	539.56 ± 39.97	< 0.001	None
Corneal volume (mm³)	62.83 ± 3.47	61.42 ± 3.44	60.76 ± 3.15	58.93 ± 4.31	< 0.001	None
Anterior chamber volume (mm³)	189.95 ± 32.90	184.71 ± 36.98	138.39 ± 33.30	134.42 ± 42.11	< 0.001	None
Anterior chamber depth (mm)	3.17 ± 0.37	3.22 ± 0.40	2.80 ± 0.49	2.87 ± 0.60	< 0.001	None

Note: Descriptive summaries use the right eye only. Inferential mixed-effects models include both eyes with a participant-level random intercept. When age group interacted significantly with sex or laterality, the overall age-group p value was not interpreted; simple effects and model-based estimates are provided in Supplementary Table S2. No pairwise age-group comparisons were retained. All p values are nominal

**Table 3:** Pearson correlation matrix for selected corneal and anterior segment parameters (right eye only)

Parameter	Anterior asphericity (Q)	Posterior asphericity (Q)	Anterior K1-flat (D)	Anterior K2-steep (D)	Anterior Kmean (D)	Anterior chamber volume (mm³)	Thinnest corneal point (µm)	Anterior chamber depth (mm)
Anterior asphericity (Q)								
Posterior asphericity (Q)	0.216 (< 0.001)							
Anterior K1-flat (D)	-0.081 (0.046)	0.069 (0.090)						
Anterior K2-steep (D)	-0.152 (< 0.001)	0.064 (0.119)	0.866 (< 0.001)					
Anterior Kmean (D)	-0.121 (0.003)	0.070 (0.087)	0.966 (< 0.001)	0.964 (< 0.001)				
Anterior chamber volume (mm³)	0.076 (0.063)	0.405 (< 0.001)	-0.121 (0.003)	-0.100 (0.014)	-0.114 (0.005)			
Thinnest corneal point (µm)	0.034 (0.407)	-0.111 (0.007)	-0.152 (< 0.001)	-0.120 (0.003)	-0.138 (< 0.001)	-0.049 (0.231)		
Anterior chamber depth (mm)	0.086 (0.035)	0.324 (< 0.001)	0.024 (0.560)	0.045 (0.271)	0.036 (0.378)	0.724 (< 0.001)	-0.062 (0.128)	

Note: Cells show Pearson correlation coefficient r (two-sided p value). Right-eye measurements were used to ensure one observation per participant (*n* = 601)

### Continuous age analysis

To characterize age-related patterns without imposing arbitrary group boundaries, age was also analyzed as a continuous variable. Age was centered at the sample mean (35.246 years) before calculating the quadratic term: age_c = age − 35.246 and age_c² = age_c × age_c. Continuous-age models included age_c, age_c², sex, laterality, age_c × sex, age_c² × sex, age_c × laterality, age_c² × laterality, and sex × laterality, with the same participant-level random intercept. Evidence of nonlinearity was based on a significant quadratic age term or quadratic age interaction. Model-based predicted trajectories with 95% confidence intervals are provided in Supplementary Figures S1–S8. Approximate turning points were calculated as − β1/(2β2) on the centered-age scale and converted back to chronological age; only turning points within the observed 3–86-year range were interpreted, and angular axis outcomes were interpreted cautiously because axis measurements are circular.

Model diagnostics, missing data, and sensitivity analysis. The source-verified final dataset contained complete age, sex, laterality, and outcome measurements for all 601 participants (1202 eyes); therefore, no imputation was required. Residual-versus-fitted and normal Q–Q plots were examined for each model. Several outcomes showed heavy-tailed residuals because of source-verified extreme observations; these observations were retained because they were confirmed in the source records and no prespecified exclusion criterion justified their removal. A post hoc sensitivity analysis excluding observations with an absolute standardized conditional residual > 3 was used only to assess robustness and did not alter the direction or statistical support of the principal anterior quadratic trajectories or the age associations of thinnest corneal point, corneal volume, anterior chamber volume, and anterior chamber depth (Supplementary Table S5). The final mixed-effects analyses were performed using Python 3.13.5 with statsmodels version 0.14.6 (MixedLM). Fixed-effect inference used two-sided asymptotic Wald tests; statsmodels MixedLM does not apply a separate denominator degrees-of-freedom approximation

### Multiple comparisons and sample size considerations

Because a large number of corneal and anterior segment parameters were evaluated and no single primary endpoint was prespecified, no across-outcome multiplicity correction was applied. Reported p values are therefore nominal and the analyses are considered exploratory and hypothesis-generating. Interpretation emphasizes effect estimates, 95% confidence intervals, consistency between categorical and continuous-age models, and robustness in sensitivity analyses rather than isolated threshold crossing.

Given the retrospective, descriptive nature of the study and the absence of a single predefined primary endpoint, no a priori sample size or power calculation was performed. The sample size was determined by the number of participants meeting the eligibility criteria during the study period. Accordingly, non-significant findings in particular should not be interpreted as definitive evidence of an absence of association.

## Results

A total of 601 participants (1202 eyes) were included. Participant-level descriptive summaries used right-eye measurements, whereas inferential analyses incorporated both eyes using mixed-effects models. Age ranged from 3 to 86 years (mean 35.25 ± 23.02 years). There were 295 male participants (49.1%) and 306 female participants (50.9%). Mean age was 35.99 ± 23.79 years in males and 34.53 ± 22.27 years in females; this difference was not statistically significant (Welch t test, *p* = 0.436).

The age-group distribution was 196 participants (32.61%) aged 3–19 years, 163 (27.12%) aged 20–39 years, 114 (18.97%) aged 40–59 years, and 128 (21.30%) aged ≥ 60 years.

Descriptive Pentacam parameters by sex are presented in Table 1. Significant sex × laterality interactions were identified for anterior K1-flat, anterior Rflat, anterior steep astigmatism axis, posterior astigmatism magnitude, posterior steep astigmatism axis, and corneal volume; therefore, a single overall sex effect was not interpreted for these outcomes. For outcomes without a significant interaction involving sex, the overall model-based sex effect is reported in Table 1. Simple sex effects for significant sex × laterality interactions are provided in Supplementary Table S3.

Descriptive Pentacam parameters across age groups are presented in Table 2. Significant overall age-group effects were observed for anterior K2-steep (*p* < 0.001), anterior Kmean (*p* < 0.001), anterior Rflat (*p* = 0.001), anterior Rsteep (*p* < 0.001), anterior Rmean (*p* < 0.001), anterior astigmatism magnitude (*p* < 0.001), anterior asphericity (*p* < 0.001), posterior K1-flat (*p* = 0.029), posterior astigmatism magnitude (*p* < 0.001), posterior asphericity (*p* < 0.001), thinnest corneal point (*p* < 0.001), corneal volume (*p* < 0.001), anterior chamber volume (*p* < 0.001), and anterior chamber depth (*p* < 0.001). Age-group effects were interaction-dependent for anterior K1-flat (age group × laterality *p* = 0.045), anterior steep astigmatism axis (*p* < 0.001), posterior K2-steep (*p* = 0.022), posterior Rsteep (*p* = 0.038), and posterior steep astigmatism axis (*p* < 0.001). For these outcomes, simple age-group effects and model-based estimates are presented by laterality in Supplementary Table S2 rather than interpreting a single overall age-group p value.

For interaction-dependent outcomes, age-group effects were supported in both eyes for anterior K1-flat (right *p* < 0.001; left *p* = 0.006), in the right eye only for posterior K2-steep (right *p* = 0.023; left *p* = 0.144) and posterior Rsteep (right *p* = 0.008; left *p* = 0.160), and were strongly laterality-dependent for the astigmatic axes. The anterior steep-axis age-group effect was present in the right eye (*p* < 0.001) but not the left eye (*p* = 0.130); posterior steep-axis orientation varied by age in both eyes (right *p* = 0.001; left *p* < 0.001), but in opposite directional patterns. These results illustrate why the corresponding overall main effects were not interpreted.

Complete fixed-effect estimates, standard errors, 95% confidence intervals, and p values for all prespecified terms in the continuous-age models are provided in Supplementary Table S1. In the continuous-age models, significant quadratic age terms were observed for anterior K1-flat (*p* = 0.022), K2-steep (*p* = 0.003), Kmean (*p* = 0.006), Rflat (*p* = 0.024), Rsteep (*p* = 0.002), Rmean (*p* = 0.006), and anterior asphericity (*p* < 0.001). The corresponding average model-based trajectories had approximate turning points at 32.2, 40.1, 36.5, 32.5, 40.2, 37.4, and 45.6 years, respectively (Supplementary Table S4 and Supplementary Figures S1–S7). Anterior steep-axis orientation also showed a significant quadratic age × laterality interaction (*p* = 0.006; Supplementary Figure S8). Average linear age associations were present for anterior astigmatism magnitude (β=−0.0061 D/year, 95% CI − 0.0091 to − 0.0031, *p* < 0.001), posterior astigmatism magnitude (β=−0.00157 D/year, 95% CI − 0.00219 to − 0.00094, *p* < 0.001), posterior asphericity (β=−0.00259/year, 95% CI − 0.00321 to − 0.00197, *p* < 0.001), thinnest corneal point (β=−0.327 μm/year, 95% CI − 0.465 to − 0.189, *p* < 0.001), and corneal volume (β=−0.0534 mm³/year, 95% CI − 0.0681 to − 0.0388, *p* < 0.001). Age slopes for anterior chamber volume and depth differed by sex (age × sex *p* = 0.038 and *p* = 0.045, respectively): the estimated ACV slopes were − 0.922 mm³/year in males and − 1.213 mm³/year in females, and the estimated ACD slopes were − 0.00470 mm/year in males and − 0.00828 mm/year in females. Posterior K2-steep, posterior Rsteep, and posterior steep-axis orientation additionally showed significant age × laterality interactions in the continuous models.

Figures 1 and 2 show raw participant-level right-eye counts and percentages and are descriptive rather than model-derived. With-the-rule (WTR) orientation was defined as a steep axis of 60°–120°, against-the-rule (ATR) as 0°–30° or 150°–180°, and oblique as 31°–59° or 121°–149°. On descriptive inspection, anterior WTR orientation became less frequent across older age groups while ATR and oblique orientations became more frequent. Posterior orientation proportions also varied across age groups. These figures are not used as evidence of statistical significance; inferential statements are based on the mixed-effects models reported in Table 2 and Supplementary Table S2.

### Correlation relationships between the parameters

Pearson correlations among selected corneal and anterior segment parameters are summarized in Table 3 and were calculated from right-eye measurements only (*n* = 601). Anterior keratometric measures were strongly correlated, including K1-flat with Kmean (*r* = 0.966, *p* < 0.001) and K2-steep with Kmean (*r* = 0.964, *p* < 0.001). Anterior chamber depth and anterior chamber volume were also strongly correlated (*r* = 0.724, *p* < 0.001).

## Discussion

Knowing the distribution of Pentacam-derived parameters in healthy corneas is important for distinguishing physiologic variation from disease and for interpreting preoperative tomography. In the final source-verified dataset, age-related change was evident but clearly non-uniform across parameters. The revised analysis uses mixed-effects models for both eyes, explicitly reports interaction-dependent effects, and supplements categorical age groups with centered continuous-age models. Consequently, the discussion below emphasizes patterns supported by the inferential models rather than raw group differences alone.

The accurate measurement of corneal parameters and knowledge of their age-related variation are particularly relevant to refractive and cataract surgery [12]. Previous studies have reported inconsistent age-related changes in keratometric values [13, 14]. In our categorical models, anterior K2 and Kmean showed significant overall age-group effects, while anterior K1 was laterality-dependent. The continuous models clarified the shape of these relationships: anterior K1, K2, and Kmean all showed significant quadratic trajectories, with model-based turning points at approximately 32, 40, and 36 years, respectively. Thus, the anterior corneal power changes in this cohort should not be described as a monotonic increase or decrease with age. Posterior keratometric behavior was less consistent: posterior K1 showed a modest categorical age-group effect, posterior K2 was laterality-dependent, and posterior Kmean did not show a robust overall age effect. These findings support a stronger and more coherent aging signal on the anterior than on the posterior keratometric surface.

Dubbelman et al. [3] reported an average anterior corneal radius of 7.79 mm and posterior radius of 6.53 mm, while other Pentacam studies have reported posterior central radii near 6.3–6.5 mm [16–18]. Our descriptive values were comparable. Anterior Rflat, Rsteep, and Rmean showed significant age-group effects and significant quadratic continuous-age trajectories, with turning points between approximately 32 and 40 years. In contrast, posterior radius measures were less uniform: posterior Rsteep showed an age-group × laterality interaction and an age × laterality interaction in the continuous model, whereas posterior Rflat and Rmean did not show robust overall age effects. Therefore, aging appears to alter anterior curvature geometry more consistently than posterior radius measures in this cohort. Previous studies have also reported age-related changes in corneal curvature and radius [19–22]. 

Posterior corneal astigmatism is clinically relevant because neglecting it can introduce error into total corneal astigmatism estimation and toric IOL planning [12, 23–25]. Prior studies have generally described an age-related shift of anterior astigmatism from WTR toward ATR orientation [21, 22, 26–30]. Our raw right-eye proportions showed the same descriptive tendency: anterior WTR orientation decreased while ATR and oblique orientations increased across age groups. However, the steep-axis models demonstrated strong laterality dependence, so the orientation graphs should not be interpreted as standalone inferential evidence. Anterior astigmatism magnitude itself showed an overall age-group effect and a small negative linear association with continuous age. Posterior astigmatism magnitude also varied with age, while posterior steep-axis orientation showed marked age × laterality effects. Taken together, the results support age-associated changes in astigmatic characteristics but argue against summarizing them as a single uniform axis shift across both eyes and both corneal surfaces.

Corneal asphericity influences retinal image quality and postoperative optical performance [9, 10, 31–35]. Previous studies have reported variable relationships between Q value and age [3, 15, 31–33]. In our revised analysis, anterior asphericity showed both a significant categorical age-group effect and a significant quadratic continuous-age trajectory, with an approximate turning point at 45.6 years. Posterior asphericity showed a strong age-group effect and became progressively more negative with continuous age. Thus, the data support age-related changes in corneal shape, but not a simple statement that the entire cornea becomes uniformly more prolate at a constant rate with age. Age-related optical changes of the human cornea have also been described [37]. 

The thinnest corneal point is an important tomographic parameter in the detection of ectatic disease [15, 38–41]. Previous Pentacam-based studies have reported limited or inconsistent age associations [36, 42]. In the source-verified final dataset, thinnest corneal point showed a significant age-group effect and a small negative continuous-age association (approximately − 0.33 μm/year). Although statistically supported, the magnitude is modest relative to interindividual variability and should not be interpreted as clinically meaningful thinning in every individual. This distinction between statistical association and individual-level clinical effect is important in normative tomography.

Anterior chamber depth (ACD) and anterior chamber volume (ACV) are clinically relevant for angle-closure risk assessment and cataract or phakic IOL planning [43–45]. Both have been reported to decrease with age, largely reflecting crystalline lens growth and anterior displacement [46–54]. In our revised models, ACV and ACD both showed highly significant age-group effects and negative continuous-age slopes. The decline was steeper in females than males for both ACV and ACD, as indicated by significant age × sex interactions. Corneal volume also decreased with age in both categorical and continuous models. These findings provide internally consistent evidence for age-associated reduction in anterior segment dimensions in this cohort.

To our knowledge, comprehensive Pentacam studies spanning childhood through older age in a healthy Turkish population remain limited. The strongest and most internally consistent age-associated findings in this cohort involved anterior corneal keratometry and radii, anterior and posterior corneal asphericity, thinnest corneal point, corneal volume, and anterior chamber dimensions. By contrast, several posterior curvature and astigmatic-axis outcomes were laterality-dependent or were supported by only one of the categorical and continuous-age formulations. Reporting both model forms therefore provides a more faithful description than reducing the data to a single linear aging trend.

This study has several limitations. First, it was retrospective and single-center and is therefore subject to selection bias. Second, although both eyes were included in inferential analyses, within-participant correlation required mixed-effects modeling; participant-level descriptive tables were therefore intentionally based on right-eye data only. Third, numerous outcomes were evaluated without an across-outcome multiplicity correction, so the analysis should be considered exploratory and findings near the significance threshold require caution. Fourth, residual diagnostics showed heavy tails for several parameters because of source-verified extreme observations. These values were retained because they were confirmed in the source records and were not excluded by prespecified criteria; post hoc residual-based sensitivity analyses supported the principal age-related findings. Fifth, steep-axis measurements are circular data, so linear mixed-model results for axis degrees and derived turning points require particularly cautious interpretation. Finally, all measurements were obtained using one Scheimpflug platform, limiting direct generalization to other devices and populations.

In conclusion, this study provides Pentacam-derived normative data for a healthy Turkish cohort and demonstrates that ocular anterior-segment aging is multidimensional rather than uniform. Anterior corneal keratometry and radii showed reproducible nonlinear age trajectories, corneal asphericity changed with age on both surfaces, and thinnest corneal point, corneal volume, ACV, and ACD decreased with increasing age. In contrast, several posterior curvature and astigmatic-axis measures depended on laterality. These results support age- and context-aware interpretation of corneal tomography in routine clinical and preoperative assessment.

## Supplementary Information

Below is the link to the electronic supplementary material.

Supplementary Material 1

## Data Availability

The datasets used and/or analyzed during the current study are available from the corresponding author on reasonable reques.
