# Supplementary material for: Age-related changes in corneal parameters in the Turkish population: a Pentacam-based study
Source: BMC Ophthalmol. 2026 Sep 23;26:593. doi: 10.1186/s12886-026-05365-9 (PMC13602622; doi:10.1186/s12886-026-05365-9)
Supplement: Supplementary file 1 [file 12886_2026_5365_MOESM1_ESM.docx]

Supplementary Material

AGE-RELATED CHANGES IN CORNEAL PARAMETERS IN THE TURKISH POPULATION: A PENTACAM-BASED STUDY. Age was centered at 35.246 years. Final mixed-effects analyses used Python 3.13.5 and statsmodels 0.14.6.

**Supplementary Table S1. Complete continuous-age mixed-effects model results**

| **Parameter** | **Fixed-effect term** | **Estimate** | **SE** | **95% CI** | **p value** |
| --- | --- | --- | --- | --- | --- |
| Anterior K1-flat (D) | Intercept | 42.589850 | 0.093692 | 42.406213 to 42.773487 | <0.001 |
| Anterior K1-flat (D) | Sex (Male; effect-coded) | -0.257766 | 0.093692 | -0.441403 to -0.074129 | 0.006 |
| Anterior K1-flat (D) | Laterality (Right; effect-coded) | -0.023335 | 0.018835 | -0.060251 to 0.013581 | 0.215 |
| Anterior K1-flat (D) | Sex × laterality | -0.022053 | 0.011724 | -0.045032 to 0.000925 | 0.060 |
| Anterior K1-flat (D) | Centered age (years) | 0.001909 | 0.002914 | -0.003803 to 0.007621 | 0.513 |
| Anterior K1-flat (D) | Centered age × sex | -0.000785 | 0.002914 | -0.006498 to 0.004927 | 0.788 |
| Anterior K1-flat (D) | Centered age × laterality | -0.000228 | 0.000587 | -0.001378 to 0.000922 | 0.698 |
| Anterior K1-flat (D) | Centered age² | 0.000318 | 0.000138 | 0.000046 to 0.000589 | 0.022 |
| Anterior K1-flat (D) | Centered age² × sex | -0.000050 | 0.000138 | -0.000321 to 0.000222 | 0.720 |
| Anterior K1-flat (D) | Centered age² × laterality | 0.000049 | 0.000028 | -0.000005 to 0.000104 | 0.076 |
| Anterior K2-steep (D) | Intercept | 43.663910 | 0.100321 | 43.467280 to 43.860540 | <0.001 |
| Anterior K2-steep (D) | Sex (Male; effect-coded) | -0.236854 | 0.100321 | -0.433484 to -0.040224 | 0.018 |
| Anterior K2-steep (D) | Laterality (Right; effect-coded) | -0.034660 | 0.018129 | -0.070193 to 0.000873 | 0.056 |
| Anterior K2-steep (D) | Sex × laterality | 0.005871 | 0.011285 | -0.016247 to 0.027988 | 0.603 |
| Anterior K2-steep (D) | Centered age (years) | -0.004366 | 0.003121 | -0.010483 to 0.001750 | 0.162 |
| Anterior K2-steep (D) | Centered age × sex | 0.000004 | 0.003121 | -0.006112 to 0.006120 | 0.999 |
| Anterior K2-steep (D) | Centered age × laterality | 0.000768 | 0.000565 | -0.000339 to 0.001875 | 0.174 |
| Anterior K2-steep (D) | Centered age² | 0.000447 | 0.000148 | 0.000157 to 0.000738 | 0.003 |
| Anterior K2-steep (D) | Centered age² × sex | -0.000022 | 0.000148 | -0.000313 to 0.000268 | 0.880 |
| Anterior K2-steep (D) | Centered age² × laterality | 0.000007 | 0.000027 | -0.000046 to 0.000059 | 0.802 |
| Anterior Kmean (D) | Intercept | 43.105771 | 0.095031 | 42.919510 to 43.292032 | <0.001 |
| Anterior Kmean (D) | Sex (Male; effect-coded) | -0.228297 | 0.095031 | -0.414558 to -0.042036 | 0.016 |
| Anterior Kmean (D) | Laterality (Right; effect-coded) | -0.008115 | 0.019813 | -0.046948 to 0.030717 | 0.682 |
| Anterior Kmean (D) | Sex × laterality | -0.018301 | 0.012332 | -0.042473 to 0.005870 | 0.138 |
| Anterior Kmean (D) | Centered age (years) | -0.000967 | 0.002956 | -0.006760 to 0.004827 | 0.744 |
| Anterior Kmean (D) | Centered age × sex | -0.000461 | 0.002956 | -0.006255 to 0.005333 | 0.876 |
| Anterior Kmean (D) | Centered age × laterality | 0.000199 | 0.000617 | -0.001011 to 0.001409 | 0.748 |
| Anterior Kmean (D) | Centered age² | 0.000388 | 0.000140 | 0.000113 to 0.000664 | 0.006 |
| Anterior Kmean (D) | Centered age² × sex | -0.000056 | 0.000140 | -0.000331 to 0.000220 | 0.692 |
| Anterior Kmean (D) | Centered age² × laterality | 0.000015 | 0.000029 | -0.000043 to 0.000072 | 0.616 |
| Anterior Rflat (mm) | Intercept | 7.932920 | 0.017387 | 7.898841 to 7.967000 | <0.001 |
| Anterior Rflat (mm) | Sex (Male; effect-coded) | 0.048109 | 0.017387 | 0.014030 to 0.082188 | 0.006 |
| Anterior Rflat (mm) | Laterality (Right; effect-coded) | 0.004120 | 0.003525 | -0.002790 to 0.011030 | 0.243 |
| Anterior Rflat (mm) | Sex × laterality | 0.004837 | 0.002194 | 0.000536 to 0.009138 | 0.028 |
| Anterior Rflat (mm) | Centered age (years) | -0.000322 | 0.000541 | -0.001382 to 0.000738 | 0.551 |
| Anterior Rflat (mm) | Centered age × sex | 0.000108 | 0.000541 | -0.000952 to 0.001168 | 0.841 |
| Anterior Rflat (mm) | Centered age × laterality | 0.000042 | 0.000110 | -0.000173 to 0.000257 | 0.702 |
| Anterior Rflat (mm) | Centered age² | -0.000058 | 0.000026 | -0.000108 to -0.000008 | 0.024 |
| Anterior Rflat (mm) | Centered age² × sex | 0.000009 | 0.000026 | -0.000041 to 0.000060 | 0.719 |
| Anterior Rflat (mm) | Centered age² × laterality | -0.000008 | 0.000005 | -0.000018 to 0.000002 | 0.120 |
| Anterior Rsteep (mm) | Intercept | 7.740084 | 0.017409 | 7.705963 to 7.774206 | <0.001 |
| Anterior Rsteep (mm) | Sex (Male; effect-coded) | 0.040430 | 0.017409 | 0.006308 to 0.074552 | 0.020 |
| Anterior Rsteep (mm) | Laterality (Right; effect-coded) | 0.004801 | 0.002652 | -0.000397 to 0.009998 | 0.070 |
| Anterior Rsteep (mm) | Sex × laterality | 0.000010 | 0.001651 | -0.003225 to 0.003245 | 0.995 |
| Anterior Rsteep (mm) | Centered age (years) | 0.000787 | 0.000542 | -0.000274 to 0.001849 | 0.146 |
| Anterior Rsteep (mm) | Centered age × sex | 0.000013 | 0.000542 | -0.001049 to 0.001074 | 0.982 |
| Anterior Rsteep (mm) | Centered age × laterality | -0.000099 | 0.000083 | -0.000261 to 0.000063 | 0.232 |
| Anterior Rsteep (mm) | Centered age² | -0.000079 | 0.000026 | -0.000130 to -0.000029 | 0.002 |
| Anterior Rsteep (mm) | Centered age² × sex | 0.000005 | 0.000026 | -0.000045 to 0.000055 | 0.844 |
| Anterior Rsteep (mm) | Centered age² × laterality | -0.000002 | 0.000004 | -0.000009 to 0.000006 | 0.677 |
| Anterior Rmean (mm) | Intercept | 7.835069 | 0.017190 | 7.801377 to 7.868761 | <0.001 |
| Anterior Rmean (mm) | Sex (Male; effect-coded) | 0.043250 | 0.017190 | 0.009558 to 0.076942 | 0.012 |
| Anterior Rmean (mm) | Laterality (Right; effect-coded) | 0.003004 | 0.003637 | -0.004123 to 0.010132 | 0.409 |
| Anterior Rmean (mm) | Sex × laterality | 0.000756 | 0.002264 | -0.003681 to 0.005193 | 0.738 |
| Anterior Rmean (mm) | Centered age (years) | 0.000297 | 0.000535 | -0.000751 to 0.001345 | 0.578 |
| Anterior Rmean (mm) | Centered age × sex | 0.000136 | 0.000535 | -0.000912 to 0.001184 | 0.799 |
| Anterior Rmean (mm) | Centered age × laterality | 0.000046 | 0.000113 | -0.000176 to 0.000268 | 0.683 |
| Anterior Rmean (mm) | Centered age² | -0.000069 | 0.000025 | -0.000119 to -0.000019 | 0.006 |
| Anterior Rmean (mm) | Centered age² × sex | 0.000006 | 0.000025 | -0.000044 to 0.000056 | 0.814 |
| Anterior Rmean (mm) | Centered age² × laterality | -0.000005 | 0.000005 | -0.000016 to 0.000005 | 0.316 |
| Anterior astigmatism (D) | Intercept | 1.066807 | 0.048668 | 0.971417 to 1.162197 | <0.001 |
| Anterior astigmatism (D) | Sex (Male; effect-coded) | 0.023962 | 0.048668 | -0.071428 to 0.119353 | 0.622 |
| Anterior astigmatism (D) | Laterality (Right; effect-coded) | -0.002627 | 0.019844 | -0.041521 to 0.036267 | 0.895 |
| Anterior astigmatism (D) | Sex × laterality | 0.022263 | 0.012352 | -0.001946 to 0.046473 | 0.071 |
| Anterior astigmatism (D) | Centered age (years) | -0.006115 | 0.001514 | -0.009082 to -0.003148 | <0.001 |
| Anterior astigmatism (D) | Centered age × sex | 0.000545 | 0.001514 | -0.002422 to 0.003512 | 0.719 |
| Anterior astigmatism (D) | Centered age × laterality | 0.000785 | 0.000618 | -0.000427 to 0.001997 | 0.204 |
| Anterior astigmatism (D) | Centered age² | 0.000133 | 0.000072 | -0.000008 to 0.000274 | 0.065 |
| Anterior astigmatism (D) | Centered age² × sex | 0.000026 | 0.000072 | -0.000115 to 0.000167 | 0.716 |
| Anterior astigmatism (D) | Centered age² × laterality | -0.000037 | 0.000029 | -0.000094 to 0.000021 | 0.211 |
| Anterior steep astigmatism axis (°) | Intercept | 91.236088 | 1.494580 | 88.306710 to 94.165465 | <0.001 |
| Anterior steep astigmatism axis (°) | Sex (Male; effect-coded) | -0.942897 | 1.494580 | -3.872274 to 1.986481 | 0.528 |
| Anterior steep astigmatism axis (°) | Laterality (Right; effect-coded) | 2.119517 | 1.487737 | -0.796447 to 5.035481 | 0.154 |
| Anterior steep astigmatism axis (°) | Sex × laterality | 2.434019 | 0.926048 | 0.618965 to 4.249074 | 0.009 |
| Anterior steep astigmatism axis (°) | Centered age (years) | -0.094797 | 0.046490 | -0.185918 to -0.003676 | 0.041 |
| Anterior steep astigmatism axis (°) | Centered age × sex | 0.017059 | 0.046490 | -0.074062 to 0.108181 | 0.714 |
| Anterior steep astigmatism axis (°) | Centered age × laterality | -0.219811 | 0.046359 | -0.310674 to -0.128948 | <0.001 |
| Anterior steep astigmatism axis (°) | Centered age² | -0.004018 | 0.002209 | -0.008348 to 0.000312 | 0.069 |
| Anterior steep astigmatism axis (°) | Centered age² × sex | 0.004209 | 0.002209 | -0.000120 to 0.008539 | 0.057 |
| Anterior steep astigmatism axis (°) | Centered age² × laterality | -0.006051 | 0.002202 | -0.010367 to -0.001736 | 0.006 |
| Anterior asphericity (Q) | Intercept | -0.302081 | 0.008645 | -0.319026 to -0.285136 | <0.001 |
| Anterior asphericity (Q) | Sex (Male; effect-coded) | 0.013228 | 0.008645 | -0.003717 to 0.030173 | 0.126 |
| Anterior asphericity (Q) | Laterality (Right; effect-coded) | 0.003139 | 0.004371 | -0.005428 to 0.011706 | 0.473 |
| Anterior asphericity (Q) | Sex × laterality | 0.003024 | 0.002721 | -0.002309 to 0.008357 | 0.266 |
| Anterior asphericity (Q) | Centered age (years) | 0.001543 | 0.000269 | 0.001016 to 0.002071 | <0.001 |
| Anterior asphericity (Q) | Centered age × sex | 0.000213 | 0.000269 | -0.000314 to 0.000740 | 0.429 |
| Anterior asphericity (Q) | Centered age × laterality | 0.000203 | 0.000136 | -0.000064 to 0.000470 | 0.136 |
| Anterior asphericity (Q) | Centered age² | -0.000075 | 0.000013 | -0.000100 to -0.000050 | <0.001 |
| Anterior asphericity (Q) | Centered age² × sex | -0.000003 | 0.000013 | -0.000028 to 0.000022 | 0.798 |
| Anterior asphericity (Q) | Centered age² × laterality | -0.000005 | 0.000006 | -0.000017 to 0.000008 | 0.459 |
| Posterior K1-flat (D) | Intercept | -6.151793 | 0.015703 | -6.182570 to -6.121016 | <0.001 |
| Posterior K1-flat (D) | Sex (Male; effect-coded) | 0.045015 | 0.015703 | 0.014238 to 0.075792 | 0.004 |
| Posterior K1-flat (D) | Laterality (Right; effect-coded) | 0.000829 | 0.005210 | -0.009384 to 0.011041 | 0.874 |
| Posterior K1-flat (D) | Sex × laterality | 0.003481 | 0.003243 | -0.002875 to 0.009838 | 0.283 |
| Posterior K1-flat (D) | Centered age (years) | -0.000846 | 0.000488 | -0.001804 to 0.000111 | 0.083 |
| Posterior K1-flat (D) | Centered age × sex | -0.000084 | 0.000488 | -0.001041 to 0.000874 | 0.864 |
| Posterior K1-flat (D) | Centered age × laterality | 0.000055 | 0.000162 | -0.000263 to 0.000373 | 0.734 |
| Posterior K1-flat (D) | Centered age² | -0.000004 | 0.000023 | -0.000050 to 0.000041 | 0.848 |
| Posterior K1-flat (D) | Centered age² × sex | -0.000011 | 0.000023 | -0.000057 to 0.000034 | 0.623 |
| Posterior K1-flat (D) | Centered age² × laterality | -0.000004 | 0.000008 | -0.000019 to 0.000011 | 0.622 |
| Posterior K2-steep (D) | Intercept | -6.502970 | 0.017869 | -6.537992 to -6.467947 | <0.001 |
| Posterior K2-steep (D) | Sex (Male; effect-coded) | 0.039531 | 0.017869 | 0.004509 to 0.074554 | 0.027 |
| Posterior K2-steep (D) | Laterality (Right; effect-coded) | 0.012119 | 0.003986 | 0.004307 to 0.019930 | 0.002 |
| Posterior K2-steep (D) | Sex × laterality | -0.004202 | 0.002481 | -0.009065 to 0.000660 | 0.090 |
| Posterior K2-steep (D) | Centered age (years) | 0.000764 | 0.000556 | -0.000325 to 0.001854 | 0.169 |
| Posterior K2-steep (D) | Centered age × sex | -0.000004 | 0.000556 | -0.001093 to 0.001086 | 0.995 |
| Posterior K2-steep (D) | Centered age × laterality | 0.000294 | 0.000124 | 0.000051 to 0.000538 | 0.018 |
| Posterior K2-steep (D) | Centered age² | 0.000012 | 0.000026 | -0.000040 to 0.000064 | 0.646 |
| Posterior K2-steep (D) | Centered age² × sex | -0.000011 | 0.000026 | -0.000063 to 0.000041 | 0.679 |
| Posterior K2-steep (D) | Centered age² × laterality | -0.000003 | 0.000006 | -0.000014 to 0.000009 | 0.647 |
| Posterior Kmean (D) | Intercept | -6.324977 | 0.015928 | -6.356197 to -6.293758 | <0.001 |
| Posterior Kmean (D) | Sex (Male; effect-coded) | 0.042434 | 0.015928 | 0.011214 to 0.073654 | 0.008 |
| Posterior Kmean (D) | Laterality (Right; effect-coded) | 0.011799 | 0.004155 | 0.003655 to 0.019942 | 0.005 |
| Posterior Kmean (D) | Sex × laterality | 0.000468 | 0.002586 | -0.004601 to 0.005536 | 0.856 |
| Posterior Kmean (D) | Centered age (years) | -0.000046 | 0.000495 | -0.001017 to 0.000926 | 0.927 |
| Posterior Kmean (D) | Centered age × sex | -0.000113 | 0.000495 | -0.001084 to 0.000858 | 0.819 |
| Posterior Kmean (D) | Centered age × laterality | 0.000211 | 0.000129 | -0.000043 to 0.000465 | 0.103 |
| Posterior Kmean (D) | Centered age² | 0.000009 | 0.000024 | -0.000038 to 0.000055 | 0.714 |
| Posterior Kmean (D) | Centered age² × sex | -0.000013 | 0.000024 | -0.000059 to 0.000034 | 0.592 |
| Posterior Kmean (D) | Centered age² × laterality | -0.000008 | 0.000006 | -0.000020 to 0.000004 | 0.199 |
| Posterior Rflat (mm) | Intercept | 6.508338 | 0.016734 | 6.475540 to 6.541136 | <0.001 |
| Posterior Rflat (mm) | Sex (Male; effect-coded) | 0.048220 | 0.016734 | 0.015422 to 0.081018 | 0.004 |
| Posterior Rflat (mm) | Laterality (Right; effect-coded) | 0.002302 | 0.006107 | -0.009668 to 0.014272 | 0.706 |
| Posterior Rflat (mm) | Sex × laterality | 0.005833 | 0.003801 | -0.001618 to 0.013284 | 0.125 |
| Posterior Rflat (mm) | Centered age (years) | -0.000819 | 0.000521 | -0.001839 to 0.000201 | 0.116 |
| Posterior Rflat (mm) | Centered age × sex | -0.000178 | 0.000521 | -0.001198 to 0.000843 | 0.733 |
| Posterior Rflat (mm) | Centered age × laterality | 0.000044 | 0.000190 | -0.000329 to 0.000417 | 0.816 |
| Posterior Rflat (mm) | Centered age² | 0.000003 | 0.000025 | -0.000045 to 0.000052 | 0.899 |
| Posterior Rflat (mm) | Centered age² × sex | -0.000015 | 0.000025 | -0.000063 to 0.000034 | 0.556 |
| Posterior Rflat (mm) | Centered age² × laterality | -0.000007 | 0.000009 | -0.000025 to 0.000010 | 0.422 |
| Posterior Rsteep (mm) | Intercept | 6.164251 | 0.016794 | 6.131335 to 6.197168 | <0.001 |
| Posterior Rsteep (mm) | Sex (Male; effect-coded) | 0.039633 | 0.016794 | 0.006716 to 0.072549 | 0.018 |
| Posterior Rsteep (mm) | Laterality (Right; effect-coded) | 0.014578 | 0.004050 | 0.006641 to 0.022516 | <0.001 |
| Posterior Rsteep (mm) | Sex × laterality | -0.001629 | 0.002521 | -0.006570 to 0.003312 | 0.518 |
| Posterior Rsteep (mm) | Centered age (years) | 0.000835 | 0.000522 | -0.000189 to 0.001859 | 0.110 |
| Posterior Rsteep (mm) | Centered age × sex | -0.000033 | 0.000522 | -0.001057 to 0.000991 | 0.950 |
| Posterior Rsteep (mm) | Centered age × laterality | 0.000344 | 0.000126 | 0.000097 to 0.000592 | 0.006 |
| Posterior Rsteep (mm) | Centered age² | 0.000009 | 0.000025 | -0.000040 to 0.000058 | 0.714 |
| Posterior Rsteep (mm) | Centered age² × sex | -0.000010 | 0.000025 | -0.000059 to 0.000038 | 0.680 |
| Posterior Rsteep (mm) | Centered age² × laterality | -0.000007 | 0.000006 | -0.000019 to 0.000005 | 0.259 |
| Posterior Rmean (mm) | Intercept | 6.335037 | 0.015927 | 6.303820 to 6.366254 | <0.001 |
| Posterior Rmean (mm) | Sex (Male; effect-coded) | 0.043242 | 0.015927 | 0.012024 to 0.074459 | 0.007 |
| Posterior Rmean (mm) | Laterality (Right; effect-coded) | 0.007392 | 0.003877 | -0.000206 to 0.014991 | 0.057 |
| Posterior Rmean (mm) | Sex × laterality | 0.000958 | 0.002413 | -0.003772 to 0.005688 | 0.691 |
| Posterior Rmean (mm) | Centered age (years) | -0.000022 | 0.000495 | -0.000993 to 0.000949 | 0.964 |
| Posterior Rmean (mm) | Centered age × sex | -0.000106 | 0.000495 | -0.001077 to 0.000866 | 0.831 |
| Posterior Rmean (mm) | Centered age × laterality | 0.000143 | 0.000121 | -0.000094 to 0.000379 | 0.238 |
| Posterior Rmean (mm) | Centered age² | 0.000008 | 0.000024 | -0.000038 to 0.000054 | 0.745 |
| Posterior Rmean (mm) | Centered age² × sex | -0.000012 | 0.000024 | -0.000058 to 0.000034 | 0.613 |
| Posterior Rmean (mm) | Centered age² × laterality | -0.000004 | 0.000006 | -0.000016 to 0.000007 | 0.439 |
| Posterior astigmatism (D) | Intercept | 0.346858 | 0.010311 | 0.326648 to 0.367068 | <0.001 |
| Posterior astigmatism (D) | Sex (Male; effect-coded) | 0.007106 | 0.010311 | -0.013104 to 0.027316 | 0.491 |
| Posterior astigmatism (D) | Laterality (Right; effect-coded) | -0.010828 | 0.005563 | -0.021732 to 0.000076 | 0.052 |
| Posterior astigmatism (D) | Sex × laterality | 0.007760 | 0.003463 | 0.000972 to 0.014547 | 0.025 |
| Posterior astigmatism (D) | Centered age (years) | -0.001565 | 0.000321 | -0.002194 to -0.000937 | <0.001 |
| Posterior astigmatism (D) | Centered age × sex | -0.000023 | 0.000321 | -0.000652 to 0.000606 | 0.943 |
| Posterior astigmatism (D) | Centered age × laterality | -0.000154 | 0.000173 | -0.000494 to 0.000185 | 0.373 |
| Posterior astigmatism (D) | Centered age² | -0.000016 | 0.000015 | -0.000045 to 0.000014 | 0.307 |
| Posterior astigmatism (D) | Centered age² × sex | -0.000005 | 0.000015 | -0.000035 to 0.000025 | 0.744 |
| Posterior astigmatism (D) | Centered age² × laterality | -0.000004 | 0.000008 | -0.000020 to 0.000012 | 0.604 |
| Posterior steep astigmatism axis (°) | Intercept | 90.309000 | 0.918722 | 88.508306 to 92.109695 | <0.001 |
| Posterior steep astigmatism axis (°) | Sex (Male; effect-coded) | -0.970078 | 0.918722 | -2.770773 to 0.830617 | 0.291 |
| Posterior steep astigmatism axis (°) | Laterality (Right; effect-coded) | 5.402301 | 0.914515 | 3.609851 to 7.194751 | <0.001 |
| Posterior steep astigmatism axis (°) | Sex × laterality | 1.832366 | 0.569244 | 0.716648 to 2.948084 | 0.001 |
| Posterior steep astigmatism axis (°) | Centered age (years) | -0.033751 | 0.028578 | -0.089764 to 0.022261 | 0.238 |
| Posterior steep astigmatism axis (°) | Centered age × sex | -0.016415 | 0.028578 | -0.072427 to 0.039598 | 0.566 |
| Posterior steep astigmatism axis (°) | Centered age × laterality | 0.175873 | 0.028497 | 0.120019 to 0.231727 | <0.001 |
| Posterior steep astigmatism axis (°) | Centered age² | -0.000065 | 0.001358 | -0.002726 to 0.002596 | 0.962 |
| Posterior steep astigmatism axis (°) | Centered age² × sex | 0.000719 | 0.001358 | -0.001942 to 0.003381 | 0.596 |
| Posterior steep astigmatism axis (°) | Centered age² × laterality | -0.001659 | 0.001353 | -0.004311 to 0.000994 | 0.220 |
| Posterior asphericity (Q) | Intercept | -0.389285 | 0.010159 | -0.409196 to -0.369373 | <0.001 |
| Posterior asphericity (Q) | Sex (Male; effect-coded) | 0.007804 | 0.010159 | -0.012108 to 0.027716 | 0.442 |
| Posterior asphericity (Q) | Laterality (Right; effect-coded) | -0.003118 | 0.004817 | -0.012559 to 0.006323 | 0.517 |
| Posterior asphericity (Q) | Sex × laterality | 0.001522 | 0.002998 | -0.004355 to 0.007399 | 0.612 |
| Posterior asphericity (Q) | Centered age (years) | -0.002586 | 0.000316 | -0.003205 to -0.001967 | <0.001 |
| Posterior asphericity (Q) | Centered age × sex | 0.000352 | 0.000316 | -0.000267 to 0.000971 | 0.265 |
| Posterior asphericity (Q) | Centered age × laterality | -0.000069 | 0.000150 | -0.000363 to 0.000225 | 0.645 |
| Posterior asphericity (Q) | Centered age² | 0.000000 | 0.000015 | -0.000029 to 0.000030 | 0.989 |
| Posterior asphericity (Q) | Centered age² × sex | -0.000007 | 0.000015 | -0.000037 to 0.000022 | 0.633 |
| Posterior asphericity (Q) | Centered age² × laterality | 0.000003 | 0.000007 | -0.000011 to 0.000017 | 0.718 |
| Thinnest corneal point (µm) | Intercept | 545.676869 | 2.262329 | 541.242705 to 550.111033 | <0.001 |
| Thinnest corneal point (µm) | Sex (Male; effect-coded) | 0.464048 | 2.262329 | -3.970116 to 4.898212 | 0.837 |
| Thinnest corneal point (µm) | Laterality (Right; effect-coded) | -1.078752 | 0.588942 | -2.233079 to 0.075575 | 0.067 |
| Thinnest corneal point (µm) | Sex × laterality | 0.408038 | 0.366590 | -0.310478 to 1.126554 | 0.266 |
| Thinnest corneal point (µm) | Centered age (years) | -0.327217 | 0.070372 | -0.465147 to -0.189288 | <0.001 |
| Thinnest corneal point (µm) | Centered age × sex | 0.080636 | 0.070372 | -0.057293 to 0.218565 | 0.252 |
| Thinnest corneal point (µm) | Centered age × laterality | -0.005560 | 0.018352 | -0.041530 to 0.030409 | 0.762 |
| Thinnest corneal point (µm) | Centered age² | 0.005352 | 0.003344 | -0.001202 to 0.011906 | 0.109 |
| Thinnest corneal point (µm) | Centered age² × sex | 0.002144 | 0.003344 | -0.004410 to 0.008697 | 0.521 |
| Thinnest corneal point (µm) | Centered age² × laterality | 0.000751 | 0.000872 | -0.000957 to 0.002459 | 0.389 |
| Corneal volume (mm³) | Intercept | 61.406137 | 0.240937 | 60.933901 to 61.878373 | <0.001 |
| Corneal volume (mm³) | Sex (Male; effect-coded) | -0.189128 | 0.240937 | -0.661364 to 0.283108 | 0.432 |
| Corneal volume (mm³) | Laterality (Right; effect-coded) | -0.038340 | 0.067380 | -0.170405 to 0.093725 | 0.569 |
| Corneal volume (mm³) | Sex × laterality | 0.084320 | 0.041941 | 0.002116 to 0.166524 | 0.044 |
| Corneal volume (mm³) | Centered age (years) | -0.053446 | 0.007495 | -0.068135 to -0.038756 | <0.001 |
| Corneal volume (mm³) | Centered age × sex | 0.004650 | 0.007495 | -0.010040 to 0.019339 | 0.535 |
| Corneal volume (mm³) | Centered age × laterality | -0.002078 | 0.002100 | -0.006193 to 0.002038 | 0.322 |
| Corneal volume (mm³) | Centered age² | -0.000107 | 0.000356 | -0.000805 to 0.000591 | 0.763 |
| Corneal volume (mm³) | Centered age² × sex | 0.000316 | 0.000356 | -0.000382 to 0.001014 | 0.375 |
| Corneal volume (mm³) | Centered age² × laterality | -0.000194 | 0.000100 | -0.000389 to 0.000002 | 0.052 |
| Anterior chamber volume (mm³) | Intercept | 168.541667 | 2.258083 | 164.115825 to 172.967509 | <0.001 |
| Anterior chamber volume (mm³) | Sex (Male; effect-coded) | 7.911108 | 2.258083 | 3.485266 to 12.336950 | <0.001 |
| Anterior chamber volume (mm³) | Laterality (Right; effect-coded) | -0.377397 | 0.619031 | -1.590698 to 0.835905 | 0.542 |
| Anterior chamber volume (mm³) | Sex × laterality | -0.464304 | 0.385319 | -1.219529 to 0.290921 | 0.228 |
| Anterior chamber volume (mm³) | Centered age (years) | -1.067424 | 0.070240 | -1.205094 to -0.929753 | <0.001 |
| Anterior chamber volume (mm³) | Centered age × sex | 0.145615 | 0.070240 | 0.007945 to 0.283286 | 0.038 |
| Anterior chamber volume (mm³) | Centered age × laterality | 0.003250 | 0.019289 | -0.034557 to 0.041057 | 0.866 |
| Anterior chamber volume (mm³) | Centered age² | -0.002008 | 0.003337 | -0.008549 to 0.004534 | 0.547 |
| Anterior chamber volume (mm³) | Centered age² × sex | -0.005165 | 0.003337 | -0.011706 to 0.001377 | 0.122 |
| Anterior chamber volume (mm³) | Centered age² × laterality | -0.000035 | 0.000916 | -0.001831 to 0.001760 | 0.969 |
| Anterior chamber depth (mm) | Intercept | 3.079437 | 0.028586 | 3.023408 to 3.135466 | <0.001 |
| Anterior chamber depth (mm) | Sex (Male; effect-coded) | 0.028066 | 0.028586 | -0.027963 to 0.084095 | 0.326 |
| Anterior chamber depth (mm) | Laterality (Right; effect-coded) | -0.001984 | 0.007675 | -0.017026 to 0.013058 | 0.796 |
| Anterior chamber depth (mm) | Sex × laterality | -0.006994 | 0.004777 | -0.016357 to 0.002369 | 0.143 |
| Anterior chamber depth (mm) | Centered age (years) | -0.006490 | 0.000889 | -0.008233 to -0.004747 | <0.001 |
| Anterior chamber depth (mm) | Centered age × sex | 0.001786 | 0.000889 | 0.000043 to 0.003529 | 0.045 |
| Anterior chamber depth (mm) | Centered age × laterality | 0.000263 | 0.000239 | -0.000206 to 0.000732 | 0.271 |
| Anterior chamber depth (mm) | Centered age² | -0.000068 | 0.000042 | -0.000150 to 0.000015 | 0.110 |
| Anterior chamber depth (mm) | Centered age² × sex | -0.000006 | 0.000042 | -0.000089 to 0.000076 | 0.881 |
| Anterior chamber depth (mm) | Centered age² × laterality | 0.000015 | 0.000011 | -0.000008 to 0.000037 | 0.199 |

*Notes: Age_c = age − 35.246 years. Age_c² was calculated after centering. Sex and laterality were effect-coded, so the centered-age coefficients represent the average trajectory across sex and laterality; interaction coefficients represent deviations from that average. All prespecified interactions were retained. P values are two-sided asymptotic Wald tests.*

Supplementary Table S2. Simple age-group effects for significant age-group × laterality interactions

| **Parameter** | **Interaction p** | **Laterality** | **Simple age-group p** | **3–19 EMM (95% CI)** | **20–39 EMM (95% CI)** | **40–59 EMM (95% CI)** | **≥60 EMM (95% CI)** |
| --- | --- | --- | --- | --- | --- | --- | --- |
| Anterior K1-flat (D) | 0.045 | Right | <0.001 | 42.826 (42.623 to 43.028) | 42.501 (42.280 to 42.723) | 42.538 (42.270 to 42.805) | 43.175 (42.924 to 43.427) |
| Anterior K1-flat (D) | 0.045 | Left | 0.006 | 42.809 (42.607 to 43.011) | 42.495 (42.274 to 42.717) | 42.651 (42.384 to 42.919) | 43.081 (42.830 to 43.333) |
| Anterior steep astigmatism axis (°) | <0.001 | Right | <0.001 | 94.394 (89.908 to 98.881) | 94.184 (89.259 to 99.109) | 92.088 (86.170 to 98.006) | 67.030 (61.458 to 72.603) |
| Anterior steep astigmatism axis (°) | <0.001 | Left | 0.130 | 87.415 (82.929 to 91.902) | 88.701 (83.776 to 93.625) | 91.175 (85.257 to 97.093) | 95.680 (90.107 to 101.252) |
| Posterior K2-steep (D) | 0.022 | Right | 0.023 | -6.534 (-6.573 to -6.496) | -6.472 (-6.515 to -6.430) | -6.453 (-6.504 to -6.402) | -6.456 (-6.504 to -6.407) |
| Posterior K2-steep (D) | 0.022 | Left | 0.144 | -6.543 (-6.582 to -6.505) | -6.483 (-6.525 to -6.440) | -6.501 (-6.552 to -6.450) | -6.486 (-6.534 to -6.438) |
| Posterior Rsteep (mm) | 0.038 | Right | 0.008 | 6.128 (6.092 to 6.165) | 6.198 (6.158 to 6.238) | 6.212 (6.164 to 6.261) | 6.210 (6.164 to 6.255) |
| Posterior Rsteep (mm) | 0.038 | Left | 0.160 | 6.124 (6.087 to 6.161) | 6.178 (6.138 to 6.218) | 6.168 (6.120 to 6.217) | 6.178 (6.133 to 6.224) |
| Posterior steep astigmatism axis (°) | <0.001 | Right | 0.001 | 91.014 (88.260 to 93.768) | 94.307 (91.285 to 97.330) | 99.295 (95.663 to 102.928) | 97.408 (93.987 to 100.828) |
| Posterior steep astigmatism axis (°) | <0.001 | Left | <0.001 | 90.996 (88.242 to 93.750) | 86.754 (83.731 to 89.777) | 83.758 (80.126 to 87.391) | 78.399 (74.979 to 81.820) |

Supplementary Table S3. Simple sex effects for significant sex × laterality interactions

| **Parameter** | **Interaction p** | **Laterality** | **Male−female estimate** | **95% CI** | **p value** |
| --- | --- | --- | --- | --- | --- |
| Anterior K1-flat (D) | 0.048 | Right | -0.6323 | -0.8692 to -0.3955 | <0.001 |
| Anterior K1-flat (D) | 0.048 | Left | -0.5399 | -0.7768 to -0.3030 | <0.001 |
| Anterior Rflat (mm) | 0.022 | Right | 0.1192 | 0.0752 to 0.1631 | <0.001 |
| Anterior Rflat (mm) | 0.022 | Left | 0.0991 | 0.0552 to 0.1431 | <0.001 |
| Anterior steep astigmatism axis (°) | 0.008 | Right | 7.9123 | 2.7076 to 13.1169 | 0.003 |
| Anterior steep astigmatism axis (°) | 0.008 | Left | -1.9624 | -7.1671 to 3.2422 | 0.460 |
| Posterior astigmatism (D) | 0.033 | Right | 0.0227 | -0.0065 to 0.0518 | 0.127 |
| Posterior astigmatism (D) | 0.033 | Left | -0.0070 | -0.0361 to 0.0222 | 0.640 |
| Posterior steep astigmatism axis (°) | 0.001 | Right | 2.7096 | -0.4850 to 5.9042 | 0.096 |
| Posterior steep astigmatism axis (°) | 0.001 | Left | -4.7412 | -7.9358 to -1.5466 | 0.004 |
| Corneal volume (mm³) | 0.050 | Right | 0.1497 | -0.4681 to 0.7676 | 0.635 |
| Corneal volume (mm³) | 0.050 | Left | -0.1807 | -0.7986 to 0.4371 | 0.566 |

Supplementary Table S4. Quadratic trajectories and approximate turning points

| **Parameter** | **Direction** | **Approximate turning point (years)** | **Quadratic/interaction p** |
| --- | --- | --- | --- |
| Anterior K1-flat (D) | U-shaped | 32.2 | 0.022 |
| Anterior K2-steep (D) | U-shaped | 40.1 | 0.003 |
| Anterior Kmean (D) | U-shaped | 36.5 | 0.006 |
| Anterior Rflat (mm) | inverted U-shaped | 32.5 | 0.024 |
| Anterior Rsteep (mm) | inverted U-shaped | 40.2 | 0.002 |
| Anterior Rmean (mm) | inverted U-shaped | 37.4 | 0.006 |
| Anterior asphericity (Q) | inverted U-shaped | 45.6 | <0.001 |
| Anterior steep astigmatism axis (°) — Right eye | inverted U-shaped | 19.6 | 0.006 |
| Anterior steep astigmatism axis (°) — Left eye | U-shaped | 4.5 | 0.006 |

*Turning points were calculated from fitted quadratic trajectories and interpreted only within the observed 3–86-year range. Axis-degree turning points are descriptive because angular measurements are circular.*

Supplementary Table S5. Residual-based sensitivity analysis

| **Parameter** | **Primary n** | **Sensitivity n** | **Primary age p** | **Sensitivity age p** | **Primary age² p** | **Sensitivity age² p** |
| --- | --- | --- | --- | --- | --- | --- |
| Anterior K1-flat (D) | 1202 | 1188 | 0.513 | 0.481 | 0.022 | 0.012 |
| Anterior K2-steep (D) | 1202 | 1192 | 0.162 | 0.149 | 0.003 | 0.001 |
| Anterior Kmean (D) | 1202 | 1192 | 0.744 | 0.645 | 0.006 | 0.004 |
| Anterior Rflat (mm) | 1202 | 1186 | 0.551 | 0.519 | 0.024 | 0.012 |
| Anterior Rsteep (mm) | 1202 | 1190 | 0.146 | 0.113 | 0.002 | <0.001 |
| Anterior Rmean (mm) | 1202 | 1193 | 0.578 | 0.656 | 0.006 | 0.003 |
| Anterior astigmatism (D) | 1202 | 1186 | <0.001 | <0.001 | 0.065 | 0.144 |
| Anterior steep astigmatism axis (°) | 1202 | 1198 | 0.041 | 0.018 | 0.069 | 0.014 |
| Anterior asphericity (Q) | 1202 | 1183 | <0.001 | <0.001 | <0.001 | <0.001 |
| Posterior K1-flat (D) | 1202 | 1186 | 0.083 | 0.165 | 0.848 | 0.269 |
| Posterior K2-steep (D) | 1202 | 1188 | 0.169 | 0.187 | 0.646 | 0.674 |
| Posterior Kmean (D) | 1202 | 1183 | 0.927 | 0.829 | 0.714 | 0.742 |
| Posterior Rflat (mm) | 1202 | 1187 | 0.116 | 0.263 | 0.899 | 0.258 |
| Posterior Rsteep (mm) | 1202 | 1185 | 0.110 | 0.140 | 0.714 | 0.677 |
| Posterior Rmean (mm) | 1202 | 1186 | 0.964 | 0.848 | 0.745 | 0.719 |
| Posterior astigmatism (D) | 1202 | 1184 | <0.001 | <0.001 | 0.307 | 0.021 |
| Posterior steep astigmatism axis (°) | 1202 | 1160 | 0.238 | 0.579 | 0.962 | 0.159 |
| Posterior asphericity (Q) | 1202 | 1177 | <0.001 | <0.001 | 0.989 | 0.902 |
| Thinnest corneal point (µm) | 1202 | 1180 | <0.001 | <0.001 | 0.109 | 0.058 |
| Corneal volume (mm³) | 1202 | 1194 | <0.001 | <0.001 | 0.763 | 0.506 |
| Anterior chamber volume (mm³) | 1202 | 1174 | <0.001 | <0.001 | 0.547 | 0.258 |
| Anterior chamber depth (mm) | 1202 | 1174 | <0.001 | <0.001 | 0.110 | 0.102 |

*Sensitivity analyses were post hoc and excluded, separately for each outcome, observations with |standardized conditional residual|>3. No observations were excluded from the primary analysis because all flagged source values were verified.*

Supplementary Figure S1. Anterior K1-flat: model-predicted marginal trajectory with 95% confidence interval.

Supplementary Figure S2. Anterior K2-steep: model-predicted marginal trajectory with 95% confidence interval.

Supplementary Figure S3. Anterior Kmean: model-predicted marginal trajectory with 95% confidence interval.

Supplementary Figure S4. Anterior Rflat: model-predicted marginal trajectory with 95% confidence interval.

Supplementary Figure S5. Anterior Rsteep: model-predicted marginal trajectory with 95% confidence interval.

Supplementary Figure S6. Anterior Rmean: model-predicted marginal trajectory with 95% confidence interval.

Supplementary Figure S7. Anterior asphericity: model-predicted marginal trajectory with 95% confidence interval.

Supplementary Figure S8. Anterior steep astigmatism axis: model-predicted trajectories stratified by laterality with 95% confidence intervals. Axis degrees are circular and should be interpreted cautiously.
